# Prediction of comorbid diseases using weighted geometric embedding of human interactome

**DOI:** 10.1186/s12920-019-0605-5

**Published:** 2019-12-30

**Authors:** Pakeeza Akram, Li Liao

**Affiliations:** 1 School of Electrical Engineering and Computer Science (SEECS), National University of Sciences and Technology (NUST), H-12, Islamabad, Pakistan; 2 Department of Computer Science, University of Delaware, Newark, USA

**Keywords:** Comorbidity, Geometric space, Embedding, Support vector machine, Random forest

## Abstract

**Background:**

Comorbidity is the phenomenon of two or more diseases occurring simultaneously not by random chance and presents great challenges to accurate diagnosis and treatment. As an effort toward better understanding the genetic causes of comorbidity, in this work, we have developed a computational method to predict comorbid diseases. Two diseases sharing common genes tend to increase their comorbidity. Previous work shows that after mapping the associated genes onto the human interactome the distance between the two disease modules (subgraphs) is correlated with comorbidity.

**Methods:**

To fully incorporate structural characteristics of interactome as features into prediction of comorbidity, our method embeds the human interactome into a high dimensional geometric space with weights assigned to the network edges and uses the projection onto different dimension to “fingerprint” disease modules. A supervised machine learning classifier is then trained to discriminate comorbid diseases versus non-comorbid diseases.

**Results:**

In cross-validation using a benchmark dataset of more than 10,000 disease pairs, we report that our model achieves remarkable performance of ROC score = 0.90 for comorbidity threshold at relative risk RR = 0 and 0.76 for comorbidity threshold at RR = 1, and significantly outperforms the previous method and the interactome generated by annotated data. To further incorporate prior knowledge pathways association with diseases, we weight the protein-protein interaction network edges according to their frequency of occurring in those pathways in such a way that edges with higher frequency will more likely be selected in the minimum spanning tree for geometric embedding. Such weighted embedding is shown to lead to further improvement of comorbid disease prediction.

**Conclusion:**

The work demonstrates that embedding the two-dimension planar graph of human interactome into a high dimensional geometric space allows for characterizing and capturing disease modules (subgraphs formed by the disease associated genes) from multiple perspectives, and hence provides enriched features for a supervised classifier to discriminate comorbid disease pairs from non-comorbid disease pairs more accurately than based on simply the module separation.

## Background

Malfunction of a gene and its products can lead to diseases. It is well studied that one gene can play multiple functions resulting in multiple diseases to a person simultaneously [1, 2]. The phenomenon of having two or more diseases in one person at a time not by random chance is known as disease comorbidity [3–5]. Disease comorbidity has adverse prognosis and intense consequences, like frequent visits and longer stays at hospitals and high mortality rate [6, 7]. For instance, it is studied that sleep apnea is the secondary cause of hypertension [8]. It is shown with a small dataset that 56% of people having sleep apnea are suffering with hypertension at the same time. Another study presented that the people with both cardiovascular disorders (CVD) and chronic kidney disease (CKD) were 35% more likely to have recurrent cardiovascular events or die than those with CVD alone [5]. Drug toxicity and intolerance is also a major problem while treating such patients as multiple drugs are incorporated to treat several disorders, where these drugs might have possible negative interaction with one another [9].

The Human Disease Network (HDN) suggest common mutant genes is the cause of disease comorbidity [10]. Disease comorbidity is also possible due to enzymes catalyzation during metabolic reactions in the metabolic network [11, 12], or disease associated rewired protein-protein-interaction (PPI) [13–15]. There are a few computational approaches that have been proposed to predict disease comorbidity. In a study PPI networks was used to locate PPIs associated with co-occurrences of diseases [16], it was found that protein localization attributes to identify comorbidity in genetic diseases [17]. Another study provided the association of phenotypically similar diseases might have connection through evolutionary associated genes [18]. Recently, comoR an effective tool has been developed to predict disease comorbidity by incorporating several existing tools into one package [3]. This package is a useful tool with a limitation that each tool work independently. For instance, one tool, ComorbidityPath, predicts disease comorbidity based on disease associated pathways only and the other tool ComorbidityOMIM only consider disease gene associated from OMIM database under certain threshold only.

More recently, another study considered each disease and its associated genes as a module, i.e., a subgraph of all the genes associated with that particular disease on the human interactome [19]. In [19], an algorithm was developed to compute so-called module separation for comorbid diseases. Module separation is the average of all pair shortest distance of genes within the disease_A_ and disease_B_. And it is found that the module separation is negatively correlated with comorbidity, in other words, high comorbid diseases tend to have closer module separation. Module separation was also demonstrated to be a useful quantity in detecting missing common genes for comorbid disease pairs [20]. Most recently, an algorithm PCID has been developed for comorbidity prediction based on integration of multi-scale data [21], which uses heterogeneous information to describe diseases, including genes, protein interactions, pathways and phenotypes. The study is focused on predicting only those diseases which co-occur with some primary disease, where the primary disease should be a well-studied and tend to be comorbid, which limit the study to a small dataset of only 73 disease pairs [21].

**Fig. 1:**
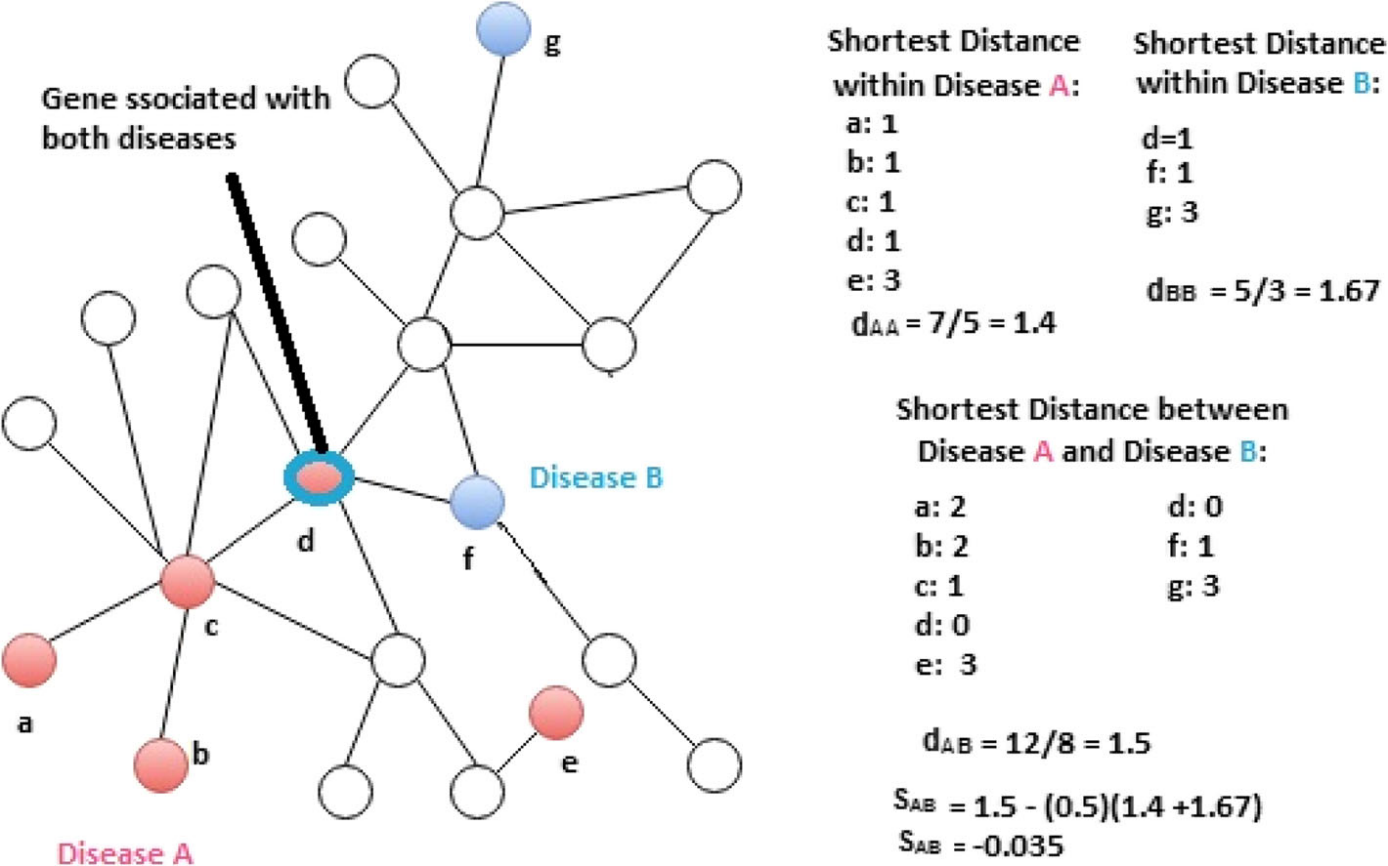
Toy example to represent two diseases as network and to calculate their module separation S_AB_

In this paper, we present a new method to predict comorbid diseases for large datasets. Our dataset comprises of 10,743 disease pair with known gene-disease association and comorbidity values. Inspired by correlation between the disease module separation S_AB_ and comorbidity in [19], our method exploits the idea of embedding the PPI network into a high dimensional geometric space in order to better characterize and incorporate interactome structural information for distinguish comorbid diseases from non-comorbid diseases. Figure 1 explains the formation of network for two diseases and formulation to calculate module separation [20]. Instead of using module separation as a means to predict comorbidity, our method first projects disease module into various dimensions to “fingerprint” the module and then trains a classifier to discriminate comorbid disease pairs from non-comorbid pairs. In 10-fold cross validation on our dataset, our method achieves a remarkable performance of ROC score = 0.9 for predicting disease pairs with relative risk RR ≥ 0 and ROC score = 0.76 for disease pairs with RR ≥ 1, which significantly outperform the performance (ROC = 0.37) from the baseline method of using the correlation between S_AB_ and RR. We also report that using a special version of weighted minimum spanning tree by assigning weights to the genes associated with a similar pathway can provide 1% improvement on the current method even on the smaller dimension then the original unweighted method. The pathway correlation is also emphasized by providing few case studies as well.

## Methods

### Overview

We considered PPI network as a graph G = (*V,E*) where *V* is a set of nodes and *E* is a set of edges. The graph is called connected if for all pairs of nodes *x*, *yϵ V* there is a path between them comprised of edges from . In general PPI networks are comprised of several subgraphs with usually one large connected component, which includes more than 90% of the information in term of proteins and their interactions. For example, we used human interactome in this study provided by [19] which has 13,460 proteins in total and the largest connected component has 13,329 proteins which comprise 99% of the total proteins in the network. In this study, we use only the largest connected component, due to the limitation of embedding in geometric space where disconnected components of a graph converted into high dimensional space may result in undefined spatial overlap.

### The embedding algorithm

The embedding algorithm used in this work is based on Multi-Dimensional Scaling (MDS) [22]. MDS is a spectral method based on eigenvalues and eigenvectors for nonlinear dimensionality reduction and uses Euclidean distance. Since human interactome is represented as a graph where coordinates of nodes are unknown, therefore an extension called isometric feature mapping based on geodesic distance is applied [23].

The basic idea of Isomap is described as follows: Given a set of *n* nodes and a distance matrix whose elements are shortest paths between all node pairs, find coordinates in a geometric space for all the nodes such that the distance matrix derived from these coordinates approximates the original geodesic distance matrix to its possible extent.

Detailed procedure for embedding task is given below:

1. Construct PPI interaction network (graph), and choose the largest connected component **G**.
2. Compute the shortest paths of all node pairs in **G** to get matrix **D**.
3. Apply the double centering to **D** and get the symmetric, positive semi-define matrix: $A=-\frac{1}{2}J{D}^2J$, ***J =*** **I** ***−*** *n*^−1^**11**^′^, where **I** is the identity matrix that has the same size as **D**; and **1** is a column vector with all one, and **1**′ is the transpose of **1**.
4. Extract the *m* largest eigenvalues λ_1_ … λ_*m*_ of **A** and the corresponding *m* eigenvectors *e*_1_ … *e*_*m*_, where *m* is the dimensions of target geometric space.
5. Then, a *m*-dimensional spatial configuration of the *n* nodes is derived from the coordinate matrix $X={E}_m{\Lambda}_m^{1/2}$, where **E**_*m*_ is the matrix with *m* eigenvectors and Λ_*m*_ is the diagonal matrix with *m* eigenvalues of **A**.

**Fig. 2:**
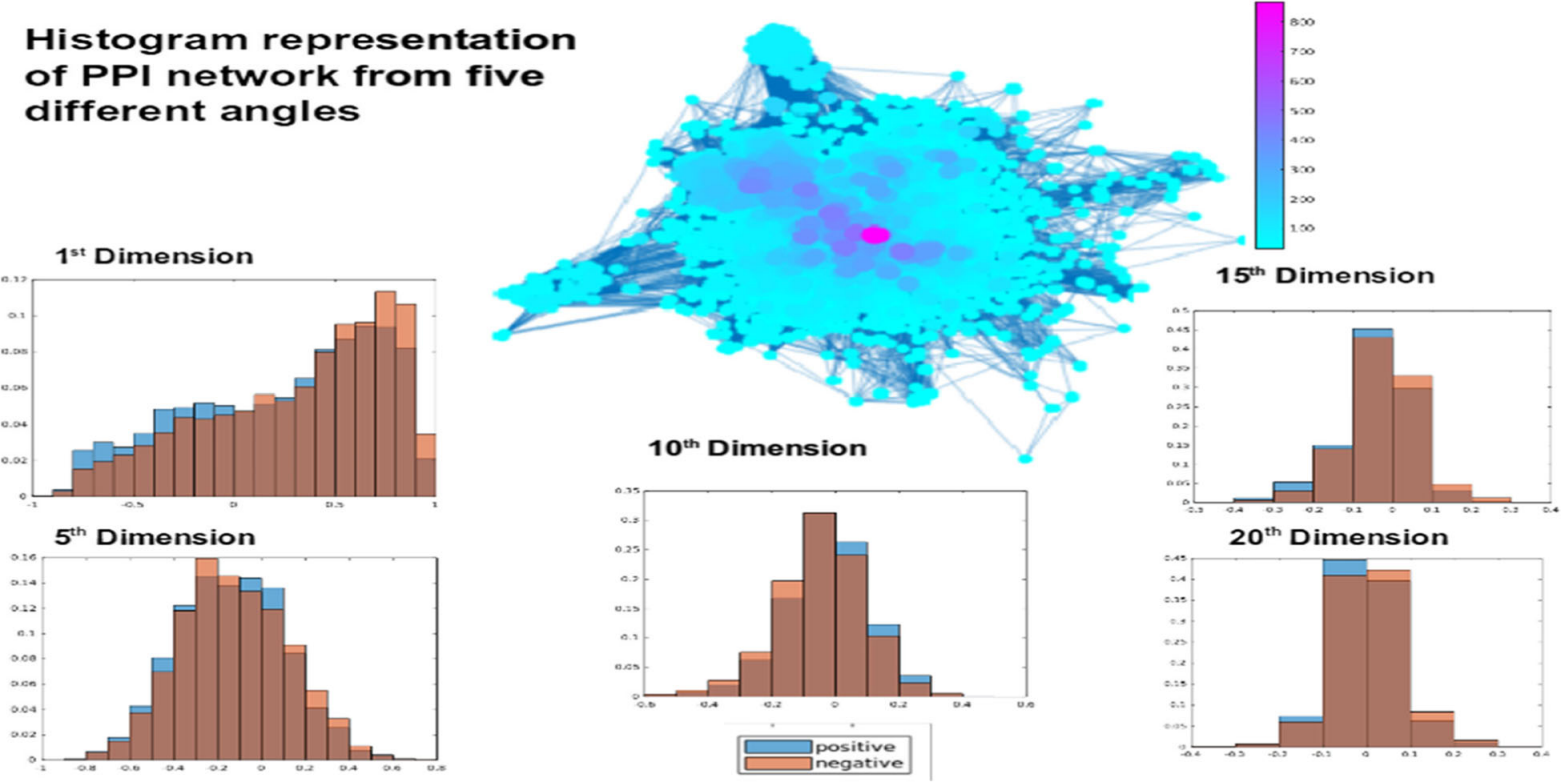
Histogram representation of PPI networks from five different angles

There are several embedding algorithms, such as Stochastic Neighbourhood Embedding (SNE) [24] and tSNE [25], Minimum Curvilinearity Embedding (MCE), non-centered MCE (ncMCE) proposed by Cannistraci et al. [26, 27]. We used the most recent MCE [27], ncMCE [26] and the method proposed by Kuchaiev et al. [28]. The Kuchaiev et al. study uses a subspace iteration to compute eigenvalues to mitigate the issue of considerable time complexity especially for larger datasets. The positive and negative examples of the comorbid disease pairs are shown in Fig. 2 from five different angles at dimension 1,5, 10, 15 and 20. The x axis of each plot is the value of the angle and the y-axis is the frequency of the angle value in the dataset.

It should be noted that the methods aforementioned are essentially based on matrix factorization. There are graph embedding algorithms that are based on other techniques, including random walks and deep learning [29, 30]. Random walk based methods approximate the graph partially using node proximity from random walks of preset length, such as DeepWalk [31] and nodd2vec [32]. Deep learning based methods use autoencoders to generate node embedding that can capture non-linearity in graphs, such as SDNE [33] and DNGR [34]. The computational complexity of these methods varies O(|V|d) for DeepWalk and node2vec, to O(|V|^2^) for ncMCE and DNGR, and to O(|V||E|) for SDNE, where |V| is the number of nodes, |E| the number of edges and d the dimension of the embedded space, see [30] for detailed comparison. The comparison of these algorithms for their pros and cons is beyond the scope of this paper. Rather, the focus of this paper is to investigate whether embedding PPI networks can help with comorbidity prediction, as compared to the existing method based on module separation.

### Disease comorbidity prediction

Our comorbidity prediction method exploits the key idea that a high dimensional geometric space provides multi facets (or angles) to capture and characterize the proteins’ relative positions in the interactome and hence makes it easier to distinguish the comorbid diseases from non- comorbid diseases by the distribution of the associated proteins on the interactome. The steps developed to implement this idea are given as follows:

1. Embed the human interactome network into a geometric space of dimension m, and extract feature vectors.
2. Choose a threshold for comorbidity
3. Train the data using a supervised learning classifier such as Support Vector Machine (SVM) or Random Forest
4. Test the model for disease comorbidity prediction.
5. Evaluate the model using several evaluation metrics

**Fig. 3:**
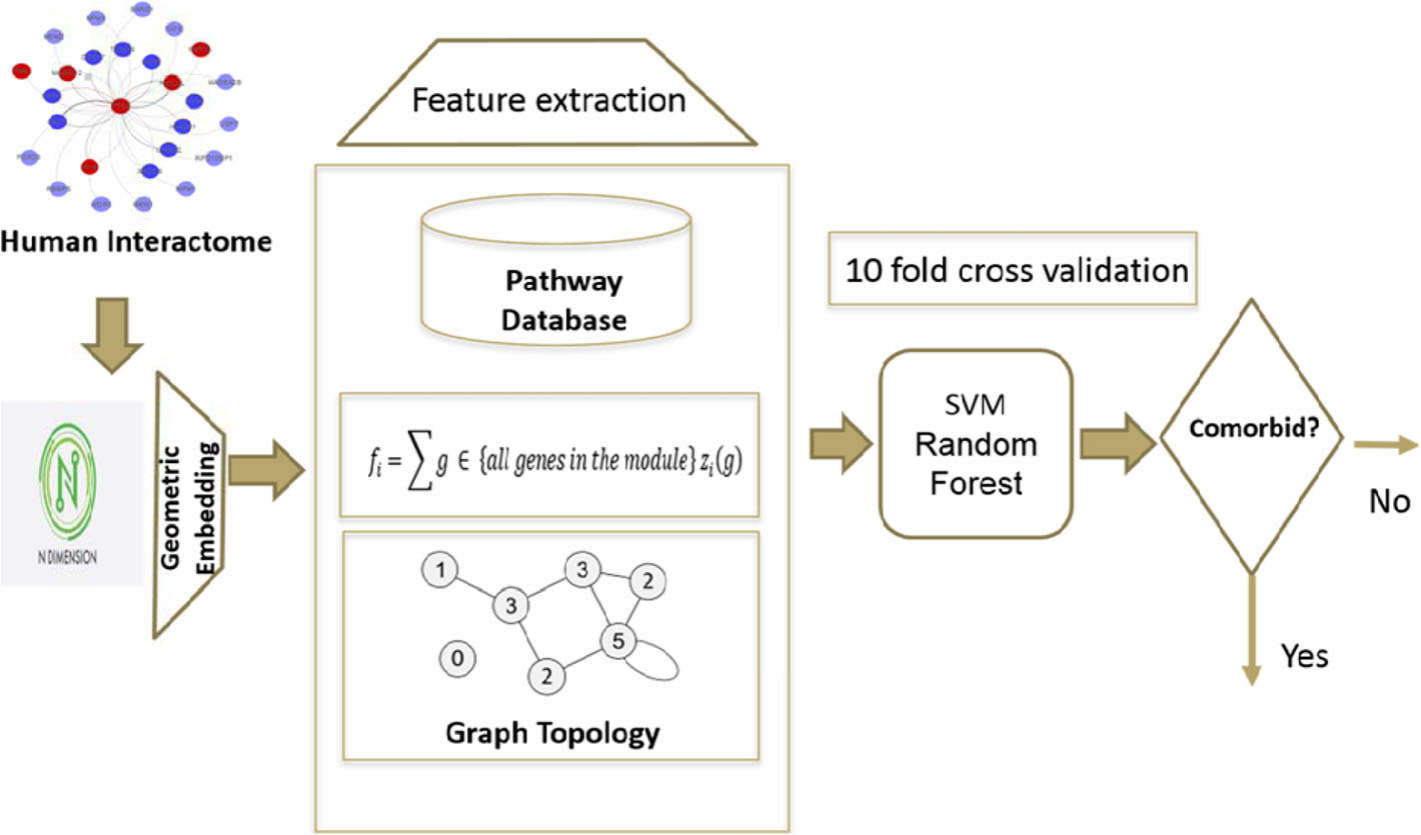
Schematic form of algorithm to predict a disease pair as comorbid or non-comorbid disease

The schematic view of the work-flow is shown is Fig. 3. The most time complex task in the pipeline is geometric embedding. We performed this task separately using a cluster Biomix at University of Delaware. It took 29.8 mins to compute geometric embedding for 20 space dimensions using the 8-core processor. The rest part was done using i7 machine with 2.56 GHz processors and 16 GB RAM. it took 10.67 mins to complete the classification after geometric embedding.

### Classification

As mentioned above, we formalize the prediction of comorbid disease as a classification problem and adopt supervised learning approach. Specifically, this is a binary classification problem where either a disease pair is comorbid or non-comorbid, corresponding to the output y of the binary classifier, namely, y = 1 for comorbid disease pair and 0 for non-comorbid disease. The classifier is to learn the actual mapping from input vector x to output: y = F(x), with a hypothesis function G (x, ɵ), where ɵ collectively represents the parameters of the classifier, for example the degree d of a polynomial kernel for SVM. The classifier is trained to minimize the empirical error.

1 $$ \min \left\{{\Sigma}_{\mathrm{i}=1\ \mathrm{to}\ \mathrm{n}}\Big\Vert \mathrm{F}\left({x}_i\right)-G\left({x}_i,\theta \right)|\right\} $$

for a set of n training examples x_i_, i = 1 to n, whose comorbid property y_i_ = F (x_i_) is known. Once the classifier is trained, it is used to make prediction / classification on unseen data, i.e., disease pair whose comorbid property is not known a priori. In this study, two powerful classifiers, Random Forest [35] and Support Vector Machines [36], are selected for this study. For SVM, 3 kernel functions were adopted and assessed: Linear, Radial Basis Function,

2 $$ {K}^G\left(x,{x}^{\prime}\right)=\exp \Big(-\gamma {\left(\left|\left|x-{x}^{\prime}\right|\right|\right)}^2/c $$

where the parameter C = 3.5 and 훾 = 1.06 and Polynomial

3 $$ {K}^P\left(x,{x}^{\prime}\right)=\left(\left\langle x,{x}^{\prime}\right\rangle \right)+1\Big){}^d $$

where the degree d = 4. These values of C, 훾 and d were optimized by using Opunity 1.1.1, a python package.

### Data and feature characterization

The dataset used in this study was adopted from [19], which consists of 10,743 disease pairs with comorbidity measured as relative risk RR based on clinical data; RR > 1 for a disease pair indicates that the diseases are diagnosed more often in the same patients that expected by chance given their individual prevalence. This comorbidity value is considered as ground truth to determine disease pair and their association in terms of comorbidity. The subset comprised of these 6270 comorbid disease pairs (PP > 1) are considered as positive examples and the rest is considered as negative non-comorbid disease pairs.

We used various values of geometric space of m for this study. Therefore, the feature vector for this study is comprised of m + 3 features in total. The feature vector for any disease pair module includes m features from the geometric space <f_1_, …, f_i_, …, f_m_>, where f_i_ is the projection of the disease module onto the *i*-th dimension, i.e., the sum of *i*-th coordinate z for all genes in the given disease module.

4 $$ {f}_i={\Sigma}_{g\in \left\{\mathrm{all}\ \mathrm{genes}\ \mathrm{in}\ \mathrm{the}\ \mathrm{disease}\ \mathrm{module}\right\}}{z}_i(g) $$

where z_i_ (g) is the *i*-th coordinate z of gene g. And the rest three features are:

1. Average degree of nodes by calculating number of edges connecting to each node. We calculated average of all the proteins associated with a disease pair.
2. Second feature is the average centrality used to measure how often each graph node appears on a shortest path between two nodes in the graph. Since there can be several shortest paths between two graph nodes s and t, the centrality of node u is:

5 $$ \mathrm{c}\left(\mathrm{u}\right)={\Sigma}_{s,t\ne \mathrm{u}}\kern0.5em {\mathrm{n}}_{st}\left(\mathrm{u}\right)/{\mathrm{N}}_{st} $$

where *n*_*st*_(*u*) is the number of shortest paths from s to t that pass-through node u, and *N*_*st*_ is the total number of shortest paths from s to t. We computed the average of all the nodes associated with both diseases taking part in disease pair under consideration.

3. The last feature is the average number of pathways associated with genes of associated disease pair. This pathway count is collected from Reactome database [37, 38]. Reactome is an open source database and contains information of about 2080 human pathways which incorporates 10374 proteins.

### Cross-validation and evaluation

To assess the prediction performance, we adopt the widely accepted cross-validation scheme. Specifically, we used 10-fold cross-validation. Given the threshold (RR = 0 or RR = 1, see the Results and discussion section), the data is split to a positive set and a negative set correspondingly, namely, with disease pairs with RR score above the threshold as positive and otherwise as negative. The positive set is then randomly split to 10 equal-sized subsets, where one set is reserved as positive test set and the rest 9 subsets are combined into a positive training set. The negative set is prepared similarly. Then, a positive train set and a negative train set are combined to form a train set to train the classifier, and a positive test set is combined with a negative test set to form a test set to evaluate the trained classifier This process is repeated 10 times, with each subset being used as test set once and the average performance from 10 runs is reported. We used some commonly used measurements to report the performance, which includes accuracy, precision, recall, F1 score, and ROC score, defined as follows.

6 $$ Recall=\frac{TP}{TP+ FN} $$

7 $$ Precision=\frac{TP}{TP+ FP} $$

8 $$ Accuracy=\frac{TP+ TN}{TP+ TN+ FN+ FP} $$

9 $$ F1=2\times \frac{Precision\times Recall}{Precision+ Recall} $$

where TP stands for true positive when a disease pair correctly predicted as comorbid, TN for true negative when a disease pair correctly predicted as non-comorbid, FP for false positive when a non-comorbid disease pair incorrectly predicted as comorbid disease pair; and FN for false negative when a comorbid disease pair is incorrectly predicted as non-comorbid disease pair.

We also evaluate the performance using receiver operating characteristic (ROC) curve and Receiver operating characteristic (ROC) score. ROC is a graphical representation that illustrates the performance of a binary classifier system. The plot is created by plotting the true positive rate (TPR) against the false positive rate (FPR) as the threshold moves down the ranked list of testing examples in descending order of the prediction score. The true-positive rate is also known as sensitivity or recall while false-positive rate is also known as (1-specificity) [39].

## Results and discussion

### Dataset

The data used for this study including the human interactome, disease gene association and comorbidity values RR is adopted from [19]. The dataset contains 10,743 disease pairs. We used comorbidity values computed and reported in [19] for the classification purpose. Comorbidity RR value ranges from 0 to < 9000 for our data. There are 6269 disease pairs with comorbidity value RR > =1, which is more than 50% of our dataset.

Among these disease pairs there are 1868 disease pairs with comorbidity value RR = 0, comprising 17% of the dataset. The other disease pairs are spread out to the max RR = 8861.6 and there are only 854 disease pair with comorbidity value > 4. In addition to setting RR = 1 as the comorbidity threshold like in Ref [19], in this study we also tested with a relaxed threshold at RR = 0, namely, any disease pairs with non-zero RR value are considered comorbid disease pairs and only these pairs with zero RR value are considered non-comorbid. So correspondingly we prepare two sets of training and testing data (Comorbidity_0 and Comorbidity_1) to evaluate the performance of our method.

### Geometric space

The first crucial task of our method is to embed the interactome into a geometric space of dimension m. We tested with different dimension space values from m = 2 to m = 50, using Kuchaiev et al. [28], MCE [27], ncMCE [26] and MDS [22] and noticed that as the dimension increases, the prediction performance ROC score roughly increases as well. The increase diminishes as m goes beyond 13 for method Kuchaiev et al. while the computational time increases drastically. For ncMCE [26] and MDS [22] the relative performance was poor. Performance of centered MCE and Kuchaiev et al. was similar and the time complexity of centered MCE is much lower. Therefore, we selected the centered MCE for finding geometric embedding for our task.

We performed evaluation comorbidity threshold RR = 1, i.e., disease pairs with RR ≥ 1 are considered as positive examples and other pairs as negative examples. We used this threshold as it was shown in [19] that comorbidity 1 is the best threshold for the classification of disease pairs into comorbid and non-comorbid diseases. In this study we considered the threshold value for comorbidity value RR = 0 and 1. The average Precision, Recall, F-measure and ROC score for each threshold is listed in Table 1.

**Table 1:** Prediction evaluation of various methods at comorbidity threshold values RR = 0 and RR = 1

	Precision	Recall	F1-measure	Accuracy	ROC
Comorbidity_0					
SVM_linear	0.68	0.83	0.75	0.83	0.56
SVM_RBF	0.90	0.90	0.89	0.90	0.90
SVM_Polynomial	0.87	0.88	0.86	0.88	0.88
Random Forest	0.86	0.86	0.83	0.86	0.89
Module Separation Sab	0.65	0.47	0.37	0.47	0.34
Comorbidity_1					
SVM_linear	0.59	0.60	0.56	0.60	0.62
SVM_RBF	0.70	0.70	0.69	0.70	0.76
SVM_Polynomial	0.68	0.68	0.67	0.68	0.72
Random Forest	0.69	0.70	0.69	0.70	0.74
Module Separation Sab	0.65	0.47	0.37	0.47	0.34

**Fig. 4:**
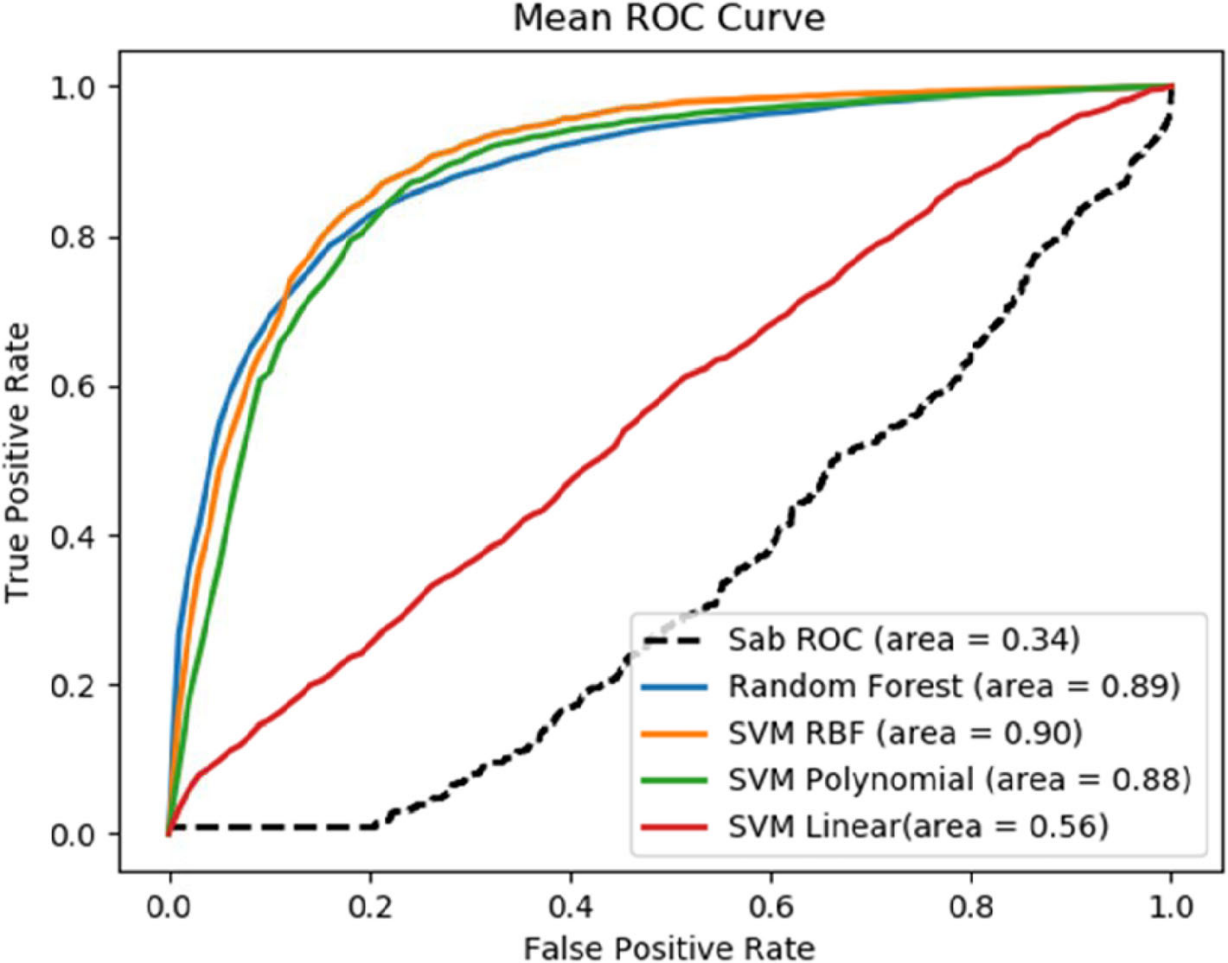
ROC Score of comorbidity prediction at RR = 0 compared with baseline

**Fig. 5:**
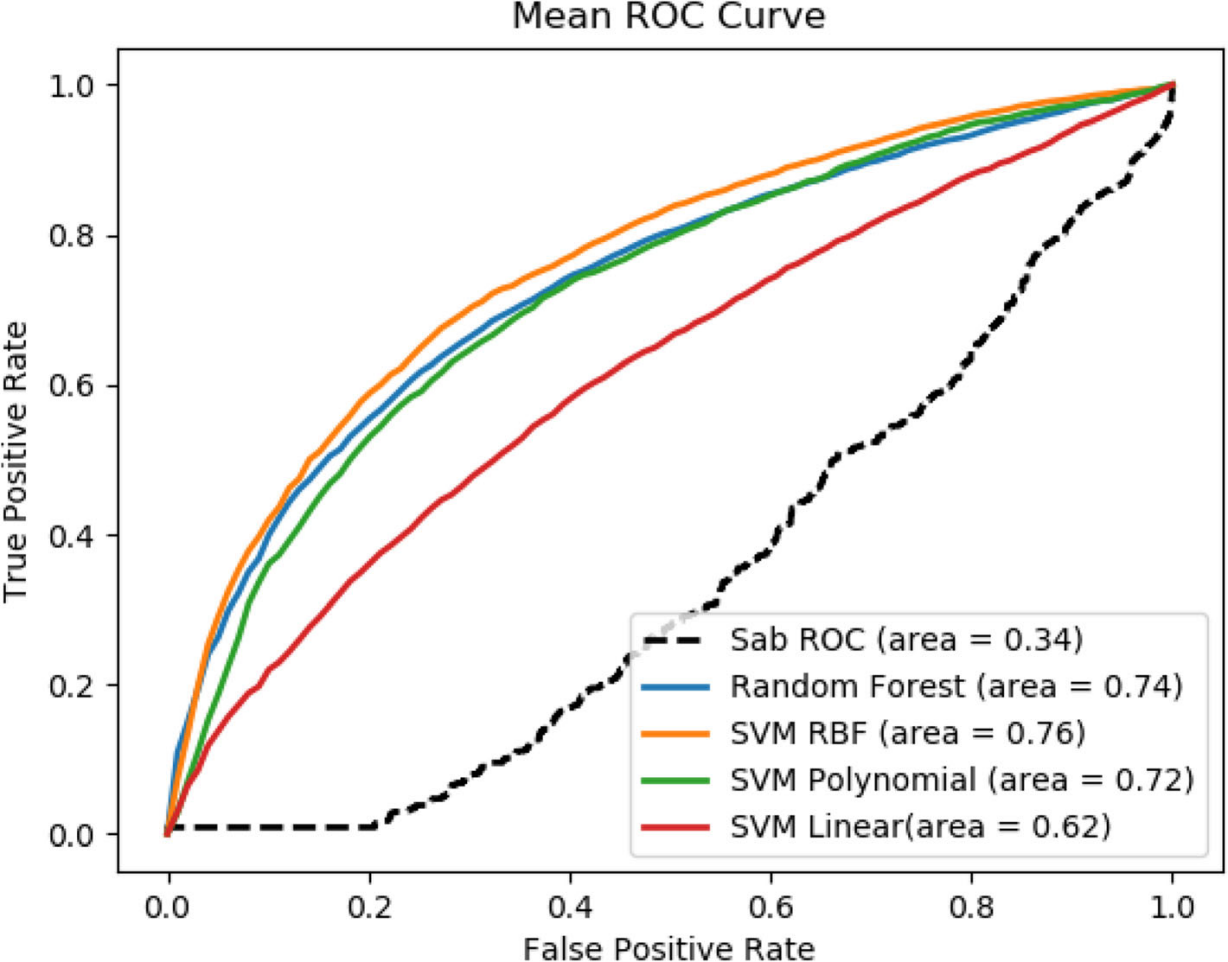
ROC Score of comorbidity prediction at RR = 1 compared with baseline

Our method significantly outperforms the baseline method, which is based on the module separation S_AB_ to predict whether a pair of disease are comorbid [19]. We compared our results with [19] since it is to our best knowledge the only study which used large amount of data for their analysis. For these variants of our method, SVM_RBF is the best performer in both datasets Comorbidity_0 (with ROC score = 0.90) and Comorbidity_1 (with ROC score = 0.76), which correspond 165% improvement and 124% improvement respectively from the baseline method. It is also noticed that, on average, better performance is achieved for the dataset Comorbidity_0, which has a more relaxed RR threshold. The ROC curve for comorbidity 0 and comorbidity 1 are shown Figs. 4 and 5 respectively. One plausible reason for SVM RBF outperforming the other selected classifiers is that SVM RBF uses a more powerful kernel function, which is capable of learning highly complex nonlinear boundary between positive data points and negative data points. Similarly, random forest strikes a good balance in discriminating positive examples from negative examples with individual decision trees and not overfitting the data with as ensemble of decision trees.

**Fig. 6:**
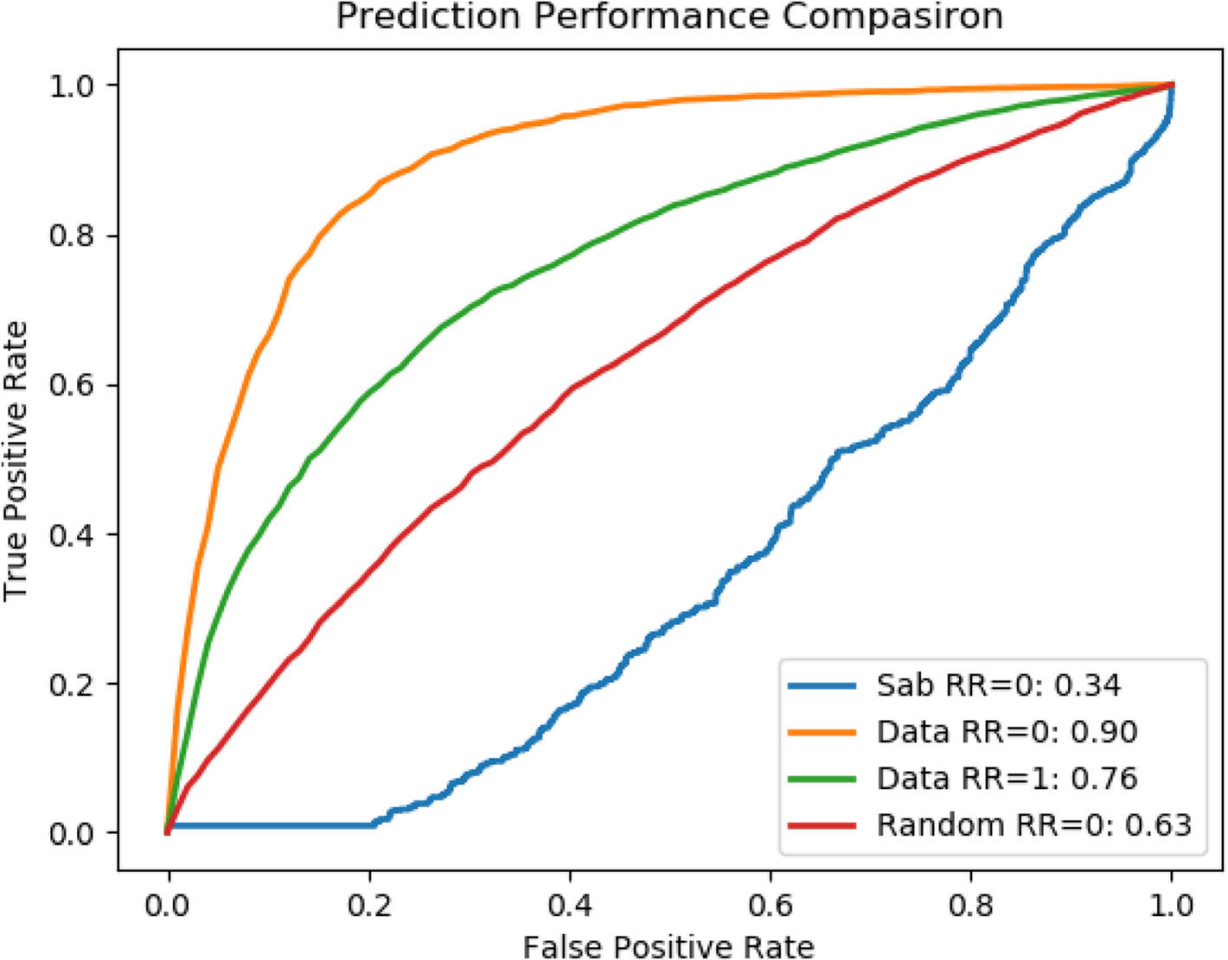
ROC Score of comorbidity prediction at RR = 0 and RR = 1 compared with random data and baseline using SVM_RBF

We also compared our results by randomizing the genes associated with a disease pair. We retained the gene count associated with each disease and the number of common genes related to a disease pair to maintain the overall topology of a disease pair sub-graph. This experiment shows that even the random data performs better than module separation method but has poor performance when compared with our approach as shown in Fig. 6. This better performance of our method is due to the spatial arrangement of proteins, which in low dimensional space captures the precise localization of proteins and its association with other proteins in a way that was not achievable by two-dimensional PPI network.

We also performed a t-test to reject the null hypothesis that performance differences are due to random fluctuation by using 10-fold-cross validation data of original data and the random data. The *p*-value of 0.0176 validates the statistical significance of our results.

Given that genes are not randomly associated with diseases and there is an underlying rewiring which connects these genes with one another to perform the proper concerned function, disruption of any gene is not damage restricted to itself but related to all the connections it made. These observations supported us to construct a network where we can observe gene related disruption easily. We created a weighted graph using the pathway information from Reactome database [37, 38]. Reactome is an open source database, and it has information of about 2080 human pathways which incorporates 10,374 proteins. We assign a weight to an edge if both the genes connected are involved in a pathway. Further, we used this weighted network to obtain the matrix D of shortest paths of all node pairs for step two of our protocol.

With the use of the weighted network, we were able to improve the prediction performance with 1% increase for 20 dimensions with *p*-value 0.93 using ROC score of 10-fold cross-validation. We suspected that might be 10-fold cross validation does not provide enough data to produce substantial results for such a small increase. Therefore, we also increased the number of cross-validation as 20, 30 and 100, the *p*-values were 0.311 and 0.29 and 0.15 respectively.

We also attempted to reduce the dimensions and observed the performance would be affected. We found that at dimension m = 13 the prediction improvement was even 1%, but the p-value was 0.009. This outcome provides a statistically significant improvement over the unweighted graph. The behavior that the performance peaks at some dimension rather than keeps going up as the dimension increases is conceivably due to the possibility that noise is also introduced. We also looked at the minimum spanning tree to see the difference in the edge selection and found that 78% of the edges are similar between the two minimum spanning tree and thus only 22% of the edges made an improvement of 1% in the performance.

### Case studies

**Fig. 7:**
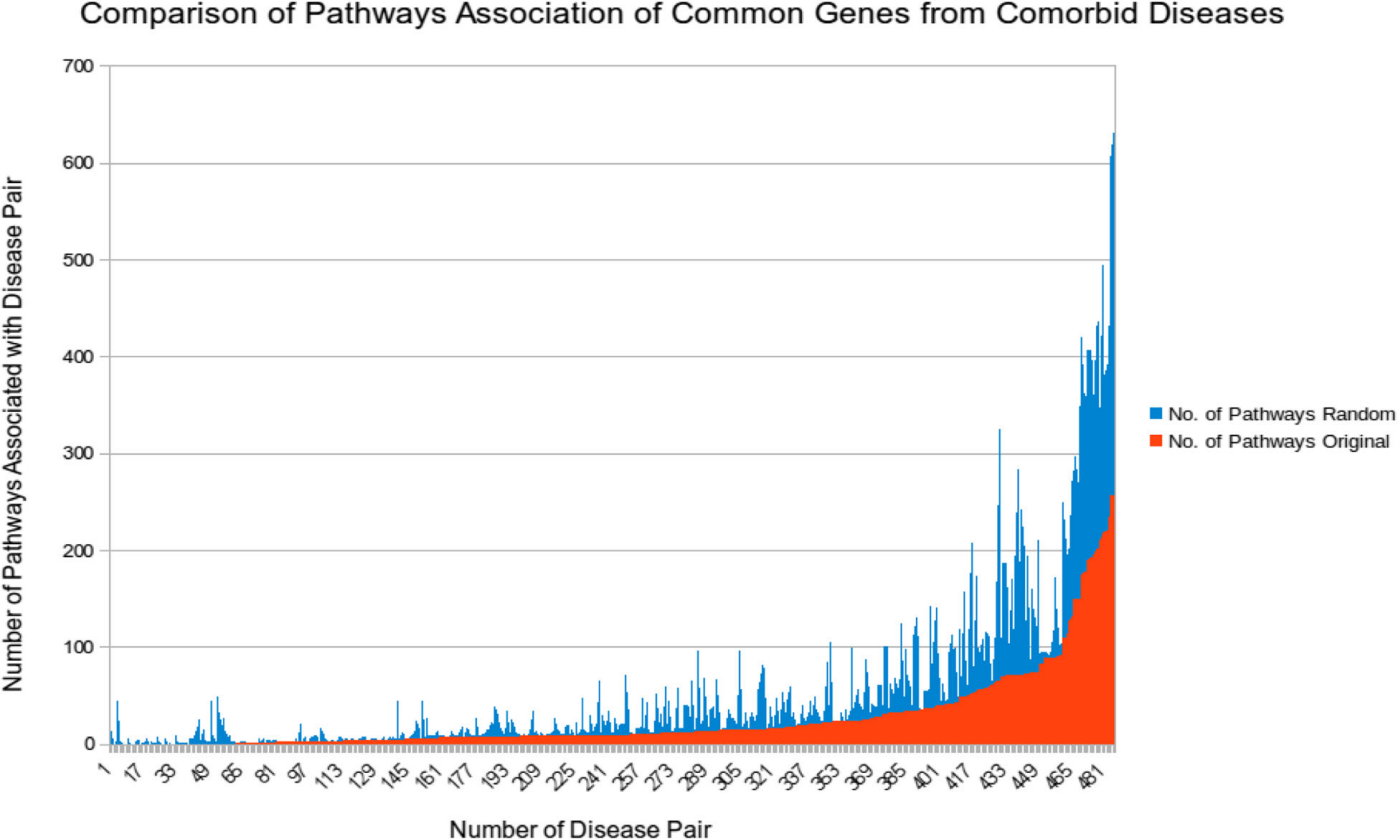
Common gene association with number of biological pathways for original and random common genes for comorbid diseases

To shed more light on how the proposed method works, case studies were conducted. We first mapped the common genes of comorbid diseases to biological pathways. We used Reactome database for this purpose. Mapping the common genes of comorbid diseases onto biological pathways shows that, as expected intuitively, as the number of common genes for comorbid disease pair increases the number of pathways associated with the disease pair also increases. To understand this relationship more quantitatively, we compared it to randomized data as a baseline. Specifically, we randomly associated common genes to disease pairs, and then observed the ratio of pathway associated with disease in the original and randomized data. Figure 7 shows the comparison histogram, displaying the frequency of pathways for common genes in the randomized vs. original data. This comparison shows that there are fewer pathways involved in comorbid diseases by real common gene association than by randomized common genes, suggesting that common genes associated with comorbid disease pair may take effect in causing both diseases simultaneously, possibly in some “coordinated” way, via disrupting fewer pathways than by random hit.

Next, we identified several disease pairs to showcase the significance and better performance ability of our protocol. We are showing two cases where module separation S_AB_ was unable to establish an association in disease pair despite a higher comorbidity value, but by projecting genes onto the higher dimension the comorbid pair was detected. It might be that these pathways associated with the disease pairs as a cause for the comorbid behavior of disease pair were properly weighted and thus resulted in an adequate embedding to the higher dimension space where the comorbid disease pairs were more easily separated from non-comorbid disease pairs. Specifically, the first disease pair shows the overlap in genes related to the two diseases. Module separation method was unable to predict this disease pair close enough to be considered as comorbid, but our method not only predict this disease pair as comorbid but also it can be seen through the case study how the pathways associated with one disease are important for the normal functioning of the other disease. The third disease pair illustrates the importance of weighted graph. In this case, both module separation and unweighted graph failed to capture comorbidity, but the weighted graph succeeded in finding a comorbid association in the disease pair, which is validated in the literature.

### Leprosy and lymphoma

**Fig. 8:**
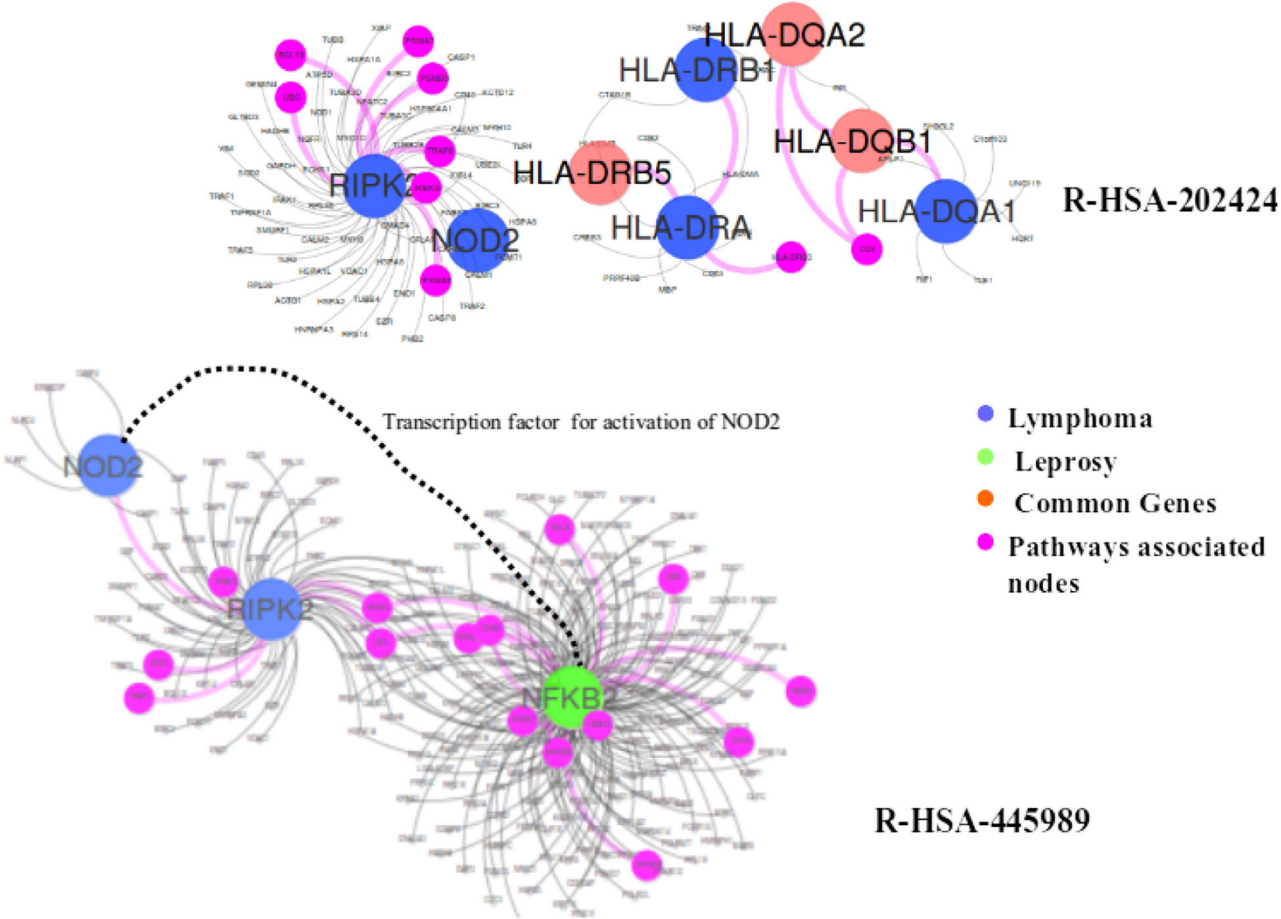
Subgraph of leprosy and lymphoma diseases

Leprosy has affected human health for decades. It is a chronic infectious disorder caused by a bacterium, Mycobacterium leprae, that affects the skin and peripheral nerves [40]. Lymphoma is a group of blood cancer developed from lymphocytes [41]. In our dataset, there are 13 genes associated with Leprosy and 24 genes related to Lymphoma. This disease pair shares three common genes HLA-DQA2, HLA-DQB1, and HLA-DRB5, and has comorbidity value RR = 1.43. while its module separation S_AB_ = 0.105 in the baseline method leads to a prediction of non-comorbidity, our method correctly classifies this disease pair as a comorbid disease pair. The common genes of the disease pair are associated with several pathways as shown in Fig. 8.

With data collection from Reactome database, we found that there are eight different pathways associated with these genes. Specifically, R-HSA-202424 has eight genes from leprosy and three genes from lymphoma taking part together. Among these genes, there are three common genes. This pathway of downstream TCR signaling has a crucial role in gene expression changes that is required for the T cell to gain full proliferative competence and to produce effector cytokines. There are three transcription factors found to play a vital role in TCR-stimulated changes in gene expression, namely NF-kB, NFAT, and AP-1.

**Fig. 9:**
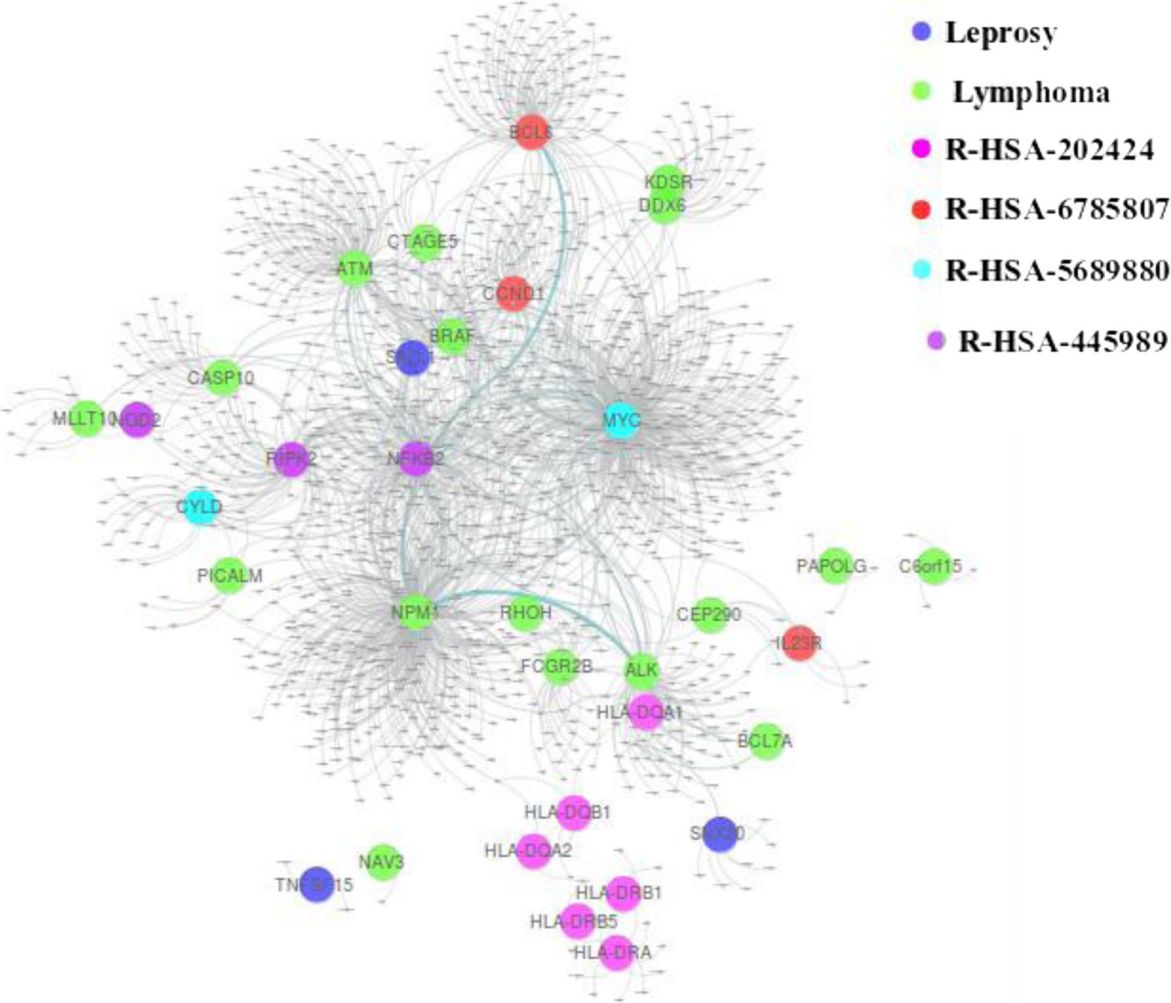
Pathway relation to genes associated with leprosy and lymphoma

We found that among these three transcription factors, NF-kB is associated with lymphoma. Interestingly, this transcription factor with two more genes related to leprosy is part of another pathway R-HSA-445989. This pathway is responsible for NFkB activation by TAK1 by phosphorylation and foractivation of IkB kinase (IKK) complex. Phosphorylation of IkB results in dissociation of NF-kappaB from the complex allowing translocation of NF-kappaB to the nucleus where it regulates gene expression. The genes associated with leprosy and pathway R-HSA-445989 have a significant role in NFkB activation which is the precursor of the TCR signaling pathway R-HSA-202424 as shown in Fig. 9.

Two more pathways: R-HSA-6785807 and R-HSA-5689880 have a common gene MYC from lymphoma and two separate genes IL23R and CYLD from leprosy associated with pathways respectively. R-HSA-6785807 also has genes BCL6, CCND1 associated with lymphoma, taking their part in the process.

**Fig. 10:**
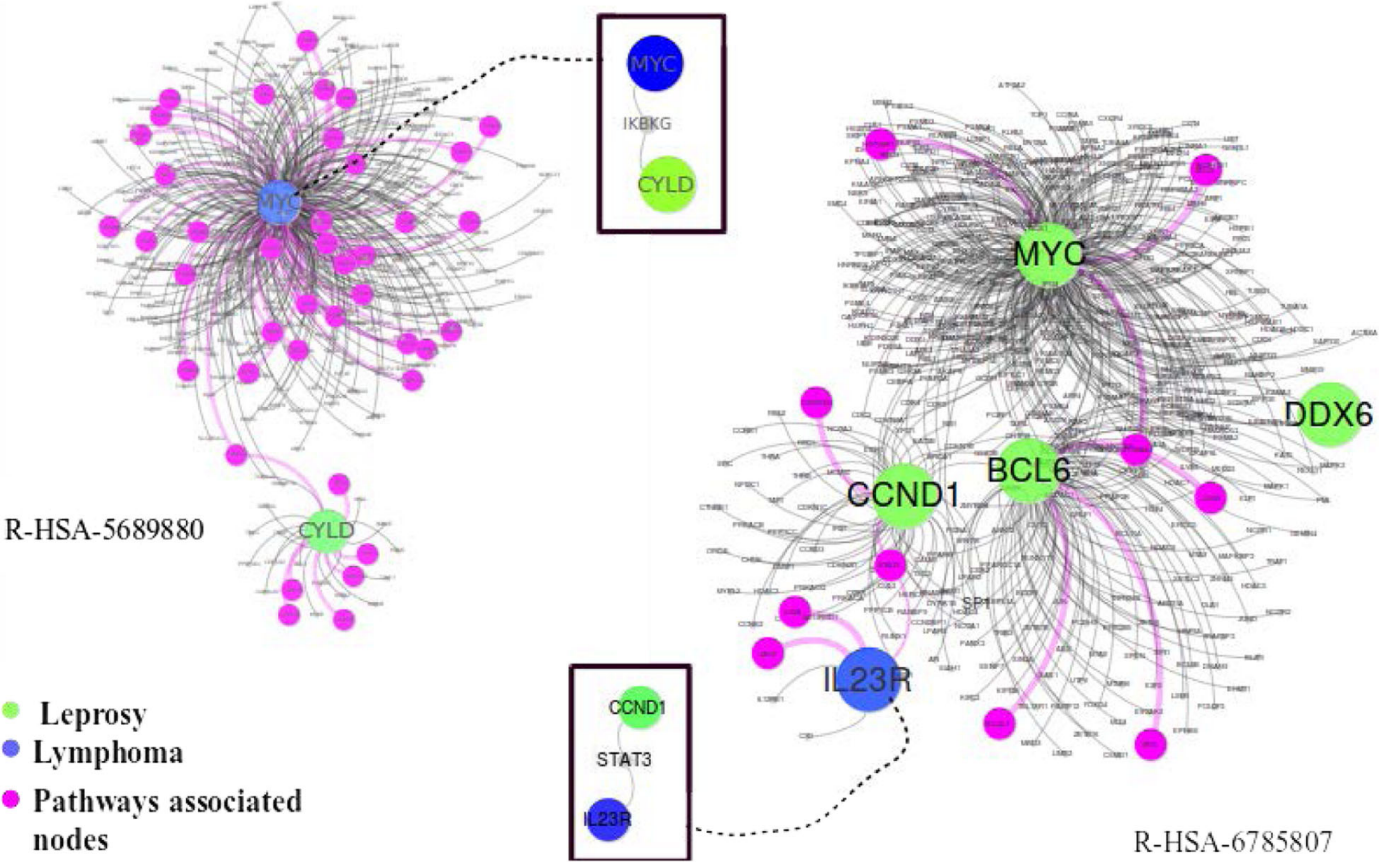
Pathway association with leprosy and lymphoma

R-HSA-5689880 is a pathway associated with Ub-specific processing proteases (USPs). They recognize their substrates by interactions of the variable regions with the substrate protein directly, or via scaffolds or adapters in multiprotein complexes. Whereas R-HSA-6785807 is Interleukin-4 and 13 signaling pathway, where Interleukin-4 (IL4) is a principal regulatory cytokine during the immune response [42]. Another interesting fact about these two pathways is that both have a direct link with gene associated with disease pair and pathway associated gene as shown in Fig. 10.

### Epilepsy and glioma

**Fig. 11:**
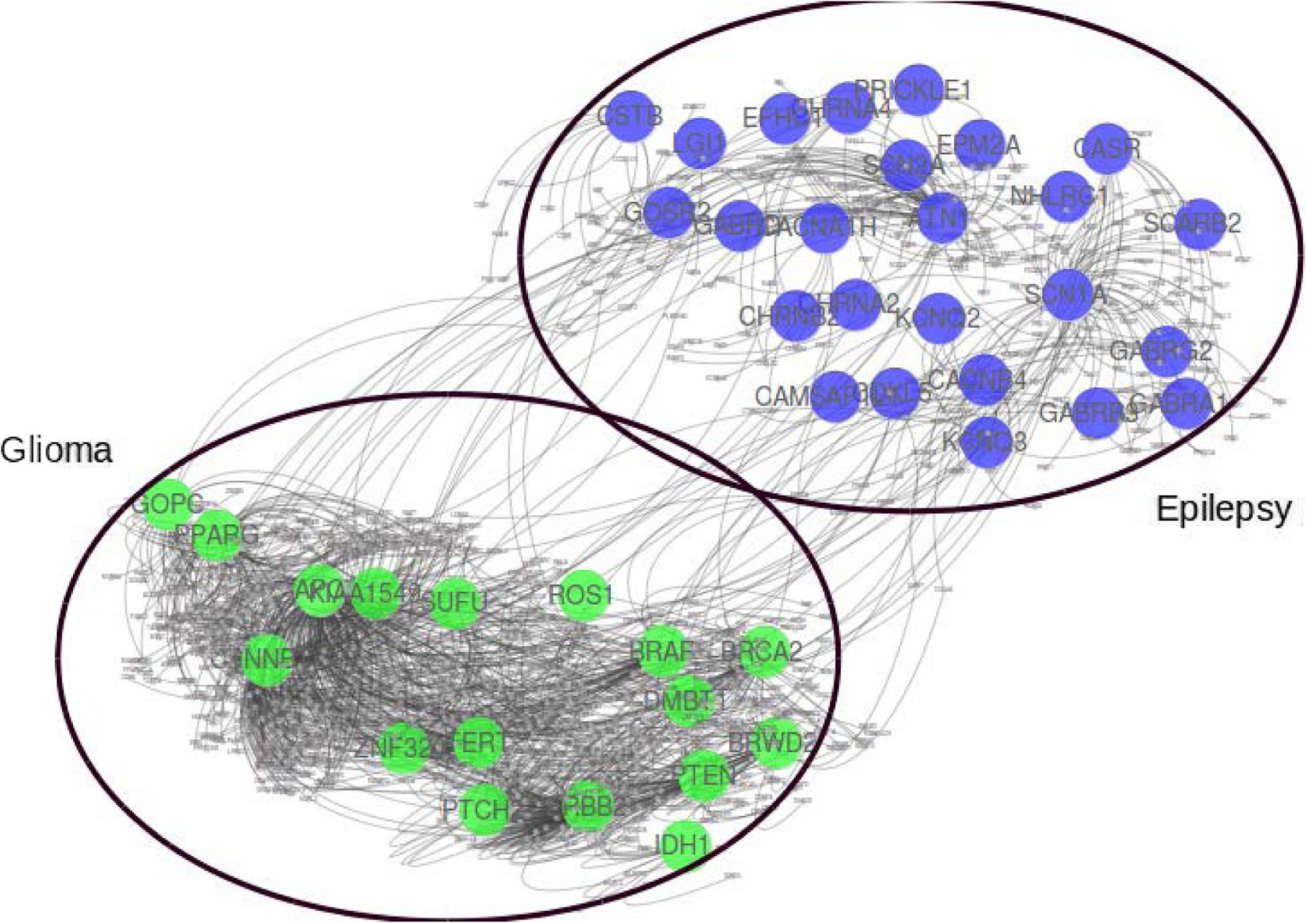
Gene Disease relation of Epilepsy and Glioma

Epilepsy is a group of neurological disorders characterized by episodes that can vary from brief to long periods of vigorous shaking. These episodes can result in physical injuries, including broken bones [43]. Glioma is a type of tumor that starts in the glial cells of the brain and spine causing 30% of all brain tumors and 80% of malignant brain tumors [44]. In our dataset, there are 25 genes associated with epilepsy and 17 genes associated with glioma. Even though both diseases are associated with the brain, there is no single common gene associated with the disease pair as shown in Fig. 11, besides having high comorbidity RR = 10.69.

Interestingly, the module separation for this disease pair is S_AB_ = 0.29, which leads to a non-comorbid prediction in the baseline method. It was also observed that our unweighted minimum spanning tree method was unable to predict it as a comorbid disease. But when we applied the weights to the genes due to their pathway association, as prescribed in the Methods section, we found that this disease pair was predicted as a comorbid disease pair. Further incorporation of pathway analysis also shows that there is a link which might cause co-occurrence of these diseases.

**Fig. 12:**
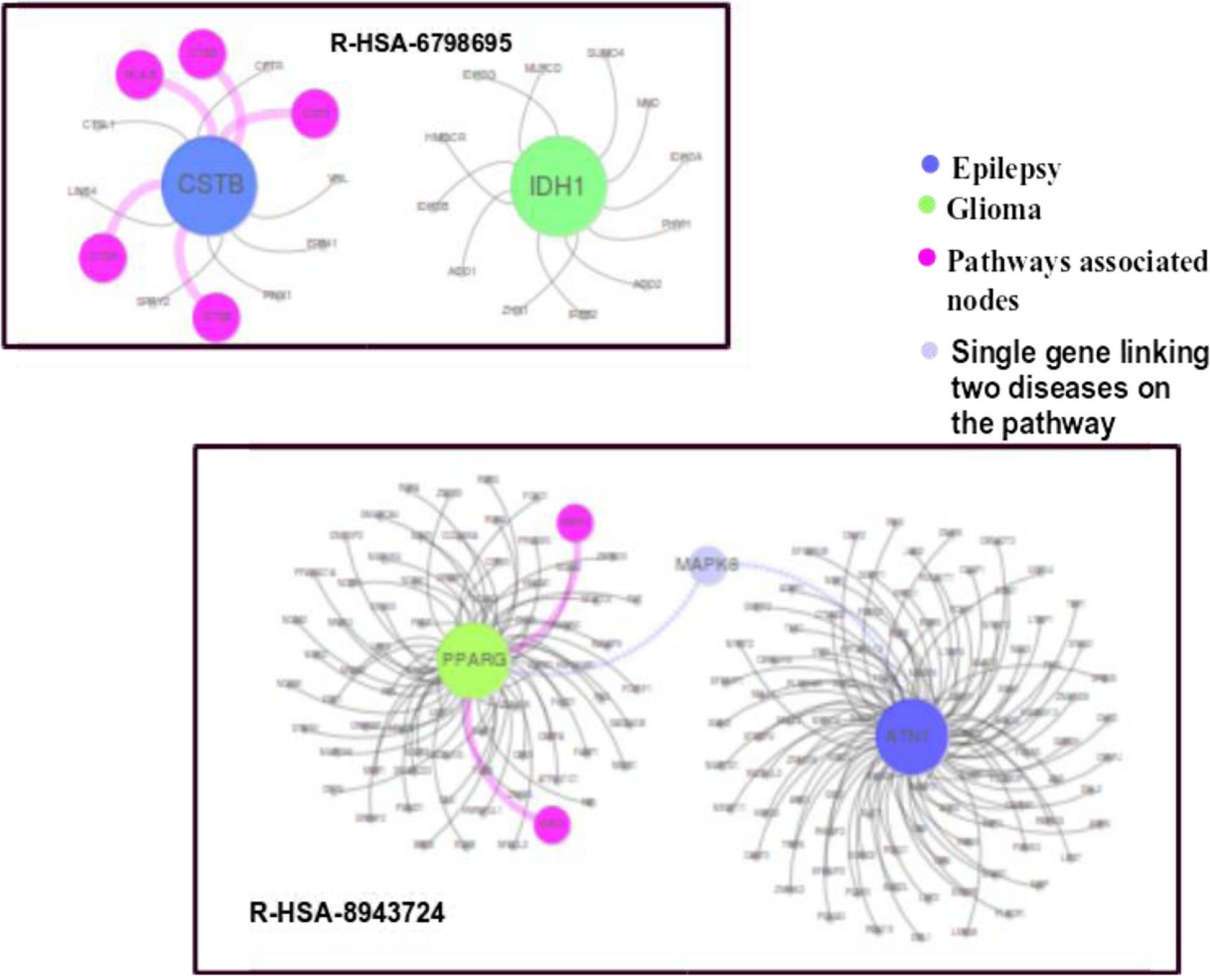
Pathways relationship with specific genes of Epilepsy and Glioma

We found that there are two pathways R-HSA-6798695 and R-HSA-8943724 associated with disease pair. R-HSA-6798695 is related to neutrophil degranulation while R-HSA-8943724 is related to regulation of PTEN gene transcription as shown in Fig. 12. PTEN gene helps in regulating cell division by keeping cells from growing and dividing too rapidly or in an uncontrolled way. On top of that, if there is any disruption in Neutrophil degranulation, it also affects the defense mechanism of the body. Literature also supports this claim that genes involved in the immune response might play a role in the pathogenesis of tumor growth as well as epileptic symptoms in patients with gliomas [45].

## Conclusion

In this work, we developed a computational method to effectively predict comorbid diseases in a large scale. While intuitively the chance for two diseases to be comorbid should go up as they have more associated genes in common, previous studies show that module separation -- how these associated genes of two diseases are distributed on the interactome plays a more important role in determining the comorbidity than does the number of common genes alone. Our key idea in this work is to embed the two-dimension planar graph of human interactome into a high dimensional geometric space so that we can characterize and capture disease modules (subgraphs formed by the disease associated genes) from multiple perspectives, and hence provide enriched features for a supervised classifier to discriminate comorbid disease pairs from non-comorbid disease pairs more accurately than based on simply the module separation. The results from cross-validation on a benchmark dataset of more 10,000 disease pairs show that our method significantly outperforms the method of using module separation for comorbidity prediction.

## Data Availability

Data was downloaded from Reference [19] at www.sciencemag.org/content/347/6224/1257601/suppl/DC1. The python code can be downloaded from the project homepage: https://www.eecis.udel.edu/~lliao/comorbidity/.

## Abbreviations

CKD: Chronic kidney disease
CVD: Cardiovascular disorders
HDN: Human Disease Network
MCE: Minimum Curvilinearity Embedding
MDS: Multidimensional Scaling
OMIM: Online Mendelian Inheritance in Man
PCID: Prediction based on integration of multi-scale data
PPI: Protein-protein Interaction
ROC: Receiver Operating Characteristics
RR: Relative risk
SVM: Support Vector Machine
